# Testis structure, duration of spermatogenesis and daily sperm production in four wild cricetid rodent species (A. cursor, A. montensis, N. lasiurus, and O. nigripes)

**DOI:** 10.1371/journal.pone.0251256

**Published:** 2021-05-20

**Authors:** Dirceu A. Cordeiro, Guilherme M. J. Costa, Luiz R. França

**Affiliations:** 1 Laboratory of Cellular Biology, Department of Morphology, Institute of Biological Sciences, Federal University of Minas Gerais—UFMG, Belo Horizonte, MG, Brazil; 2 UNINCOR, Três Corações, MG, Brazil; University of Hyderabad, INDIA

## Abstract

Although rodents represent approximately 40% of all living mammalian species, our knowledge regarding their reproductive biology is still scarce. Due to their high vulnerability to environmental changes, wild rodents have become beneficial models for ecological studies. Thus, we aimed to comparatively investigate key functional testis parameters in four sexually mature wild rodent species (*A*. *cursor*, *A*. *montensis*, *N*. *lasiurus*, and *O*. *nigripes*). These species belong to the Cricetidae family, which is the most diverse family of rodents in South America, with a total of ~120 species in Brazil. The results found for the gonadosomatic index and the sickled sperm head shape observed strongly suggest that the species here evaluated are promiscuous, prolific, and short-lived. The duration of spermatogenesis was relatively short and varied from ~35–40 days. Both the percentage of seminiferous tubules (ST) in the testis parenchyma (~95–97%) and the number of Sertoli cells (SC) (~48–70 million) per testis gram were very high, whereas a fairly good SC efficiency (~8–13 round spermatids per SC) was observed. In comparison to other mammalian species studied, particularly the rodents of the suborder Myomorpha (i.e. hamsters, rats and mice), the rodents herein investigated exhibited very high (~62–80 million) daily sperm production per testis gram. This impressive spermatogenic efficiency resulted mainly from the short duration of spermatogenesis and quite high values found for the ST percentage in the testis and the SC number per testis gram. We expect that the knowledge here obtained will help conservation programs and the proper management of wildlife.

## Introduction

Considering that wild rodents have restricted dispersion in forest regions and are highly sensitive to environmental changes, they have become valuable models for examining biodiversity alterations in the last decades [1, 2]. Besides that, this mammalian order is widely diverse and requires relatively small areas to maintain a viable population, which makes its sampling relatively easy [3]. The four species (*Akodon cursor*, *Akodon montensis*, *Necromys lasiurus*, and *Oligoryzomys nigripes*) investigated in the present study belong to the Cricetidae family, which is the most diverse family of rodents in South America and particularly in Brazil where they comprise a single neotropical Sigmodintinae subfamily with a total of 117 species and 36 genera [4].

The species *A*. *cursor* and *A*. *montensis* are insectivorous-omnivorous found in the Atlantic semi-deciduous forests [5–9], whereas *N*. *lasiurus* is a small rodent frequently found in savanna areas (also called Brazilian Cerrado) or ecotones with Atlantic Forest and feeds on insects, fruits and seeds [10]. This latter species has a smaller tail compared to its body and a distinctive periocular ring formed by light hair in each eye [4, 11]. The *O*. *nigripes* is a tiny rodent with a much larger tail compared to its body. It feeds on vegetables, commonly inhabiting open vegetation of the savanna or Atlantic Forest areas [12, 13]. As these species mentioned above inhabit a region with great mammalian diversity, they are important for the ecological interactions that occur among species [14–16].

Although often captured in areas of the Atlantic Forest and savanna, there are few studies related to the reproductive biology of these wild rodent species [17–20]. It is important to mention that these biomes have been severely fragmented and degraded by human activities for hundreds of years [21–24]. As a consequence, changes have occurred in the natural habitats and species diversity [25], which can favor generalist and opportunistic species to increase their densities and dispersion, as well as increasing the number of agricultural pests and changing the natural prevalence of zoonotic pathogens in wild reservoirs [26–28]. Moreover, the wild rodents herein investigated are classified as opportunistic species and reservoirs of zoonotic pathogens [2, 29–34]; understanding their reproductive behavior is very useful to develop conservation management strategies. For instance, the evaluation of reproductive organs in different species allows a better comprehension of distinct reproductive strategies and physiological specificities related to each species [35–42]. This evaluation is of particular interest when one considers that relatively few mammalian species have been investigated, and remarkable differences in their reproductive biology may exist [43–45]. Therefore, it cannot be assumed that reproductive mechanisms are uniform among species [46]. In this regard, the present study aimed to investigate several key morphofunctional testicular parameters and comparatively evaluate spermatogenesis in four wild Cricetidae rodent species already mentioned above.

## Material and methods

### Animals and tissue processing

The current study was performed in accordance with ethical and animal experiments regulations of the Brazilian Government (Law 11794/2008). All animal experiments were performed in strict accordance with the Guidelines for Animal Use and Experimentation. More specifically, this study was approved by the Animal Experimentation Ethics Committees of the Federal University of Minas Gerais (CEUA/UFMG, Belo Horizonte, Brazil; protocol # 94/2008). The corresponding author of this study, Dr. Luiz R. de França, who is a veterinarian (CRMV-MG #3980), gave the injections, did other necessary procedures and performed the euthanasia. During the time intervals between injection and euthanasia, the animals were maintained with ration, fruits, water ad libitum and housed in a photoperiod-controlled vivarium for wildlife species at the Department of Morphology of the Institute of Biological Sciences at the Federal University of Minas Gerais.

In the present investigation, testes from four sexually mature cricetid rodent species (*A*. *cursor*, *n* = 6; *A*. *montensis*, *n* = 9; *N*. *Lasiurus*, *n* = 13; and *O*. *nigripes*, *n* = 11) were evaluated. As these species are not seasonal [47–49], they were captured along the year in fragments of the Atlantic Forest and Brazilian savanna located in the state of Minas Gerais, Brazil (20°0′51″ S, 43°29′28″ W) during the rainy and dry seasons (S1 Table). The animals were captured using 100-wire mesh live traps placed on the ground at intervals of 15 meters along three lines spaced 20 meters apart. Traps were baited with a mixture of banana and peanut butter during four nights each month and checked in the morning.

All rodents were euthanized by anesthetic overdose [ketamine (300 mg/Kg BW) and xylazine (30 mg/Kg BW); Sigma-Aldrich, St. Louis, MO, USA]. Subsequently, testes were perfused-fixed by gravity-fed perfusion through the left ventricle with 0.9% saline and 4% buffered glutaraldehyde for 25-30min [50]. Following orchiectomy, testes were separated from the epididymis, weighed and cut longitudinally into small fragments (1-3mm thickness), which were routinely processed and embedded in glycol-methacrylate for histological and autoradiographic analysis as described below. The gonadosomatic index (GSI), which is the total testis weight divided by the BW, was also obtained for all animals.

### Thymidine injections and autoradiographic analysis

In order to estimate the duration of spermatogenesis and before orchiectomy, intratesticular injections (50μCi per testis, *n* = 2 per each species) of tritiated thymidine [thymidine (methyl-3H), specific activity 82.0 Ci mmol-1; Amersham Life Science, UK] were given near the cauda of the epididymis. Two-time intervals (1 hour and approximately three weeks) were considered after thymidine injections for *A*. *cursor*, *A*. *montensis*, and *N*. *Lasiurus*, whereas for *O*. *nigripes* these intervals were 1 hour and approximately two weeks.

For the autoradiographic analysis, unstained testis sections (4μm) were dipped in autoradiographic emulsion (Kodak NTB-2, Eastman Kodak Company; Rochester, NY) at 43–45°C. After drying for approximately 1h at 25°C, testis sections were placed in sealed black boxes and stored in a refrigerator at 4°C for approximately four weeks. Subsequently, they were developed in Kodak D-19 (Eastman Kodak Company; Rochester, NY) solution at 15°C [51] and stained with toluidine blue. Aiming to detect the most advanced germ cell type labeled at the different time periods following thymidine injections, the analyses were performed using an Olympus microscope (BX60). Cells were considered labeled when four or more thymidine grains were present over the nucleus in a low to moderate background.

### Testis morphometry

The volume densities of testicular tissue components were determined by light microscopy using a 441-intersection grid placed in the ocular. Fifteen randomly chosen fields (6,615 points) were scored for each animal at 400X magnification. The tubular diameter and seminiferous tubule epithelium height were measured at 200X magnification using an ocular micrometer. Thirty tubular profiles (round or nearly round) were chosen randomly and measured for each animal. The epithelium height was obtained in the same tubules used to determine tubular diameter. The total length of seminiferous tubules (meters) was obtained by dividing seminiferous tubule volume by the squared radius of the tubule multiplied by π [52].

### Stages of the seminiferous epithelium cycle and duration of spermatogenesis

Seminiferous epithelium cycle (SEC) stages were characterized according to the development of the acrosome system and morphology of the developing spermatid nucleus [53]. The relative stage frequencies were determined by evaluating 250 seminiferous tubule cross-sections per animal at the magnification of 400x. The seminiferous tubules analyzed were randomly chosen, and both testes were examined for each animal.

The spermatogenic cycle length was estimated based on the stage frequencies and the most advanced germ cell type labeled at different periods following thymidine injections. The total duration of spermatogenesis took into account that approximately 4.5 cycles are necessary to complete the spermatogenic process [53, 54]. Since the nuclear volume of pachytene primary spermatocytes grows markedly during the meiotic prophase, their nuclei size was used as a reference in order to precisely determine the location of the most advanced labeled germ cell at a specific SEC stage.

### Cell numbers

#### Germ and Sertoli cells

The cells present in stage VIII of the cycle were counted in ten seminiferous tubule cross-sections per animal. These counts were corrected based on the method described by Abercrombie (1946) [55], as modified by Amann (1962) [56]. Cell ratios/proportions were achieved from these corrected counts. Assuming that no significant germ cell loss occurs during spermiogenesis, the number of round spermatids was considered as the total number of produced spermatozoa [53, 57].

The Sertoli cell numbers per testis and testis gram were estimated from the Sertoli cell nucleoli number per seminiferous tubule cross-section and the obtained seminiferous tubule total length [58, 59]. This methodology is based on the fact that Sertoli cell numbers are stable in adult animals and in seminiferous tubule cross-sections in the different spermatogenic cycle stages [43, 60–62]. Daily sperm production (DSP) per testis and gram of testis (spermatogenic efficiency) were obtained according to the formula described by França (1992) [63]: DSP = Sertoli cell number per testis x the ratio of round spermatids to Sertoli cells in stage VIII x stage VIII relative frequency (%)/stage VIII duration (days).

#### Leydig cells

Leydig cell volume was obtained using the nuclear volume as well as the proportion between the nucleus and cytoplasm. For this purpose, 30 nuclei were measured per animal. Leydig cell nuclear volume was obtained using the sphere formula (4/3πR^3^, in which R = nuclear diameter/2). The proportion between the nucleus and cytoplasm was estimated, scoring 1,000 points over Leydig cells per animal using a grid placed in the ocular at 400X magnification. The number of Leydig cells per testis was estimated from the individual Leydig cell volume and the total volume occupied by these cells in the testis parenchyma.

## Results

### Biometry and morphometry

The biometric data obtained from the rodent species evaluated in the present study are shown in Table 1. The highest testicular weight was observed in *A*. *cursor*, whereas *O*. *nigripes* exhibited the smallest value for this parameter and for the gonadosomatic index that was higher in both *A*. *cursor* and *A*. *montensis*.

**Table 1:** Biometric and testis morphometric data in *A*. *cursor*, *A*. *montensis*, *N*. *Lasiurus* and *O*. *nigripes* (mean ± SEM).

Parameter			Species	
	*A*. *cursor*	*A*. *montensis*	*N*. *lasiurus*	*O*. *nigripes*
Body weight (g)	54 ± 3	37 ± 2	60 ± 3	23 ± 1
Testis weight (mg)	288 ± 27	217 ± 20	237 ± 15	63 ± 4
Gonadosomatic índex (%)	1.07 ± 0.1	1.1 ± 0.05	0.80 ± 0.04	0.5 ± 0.02
Testis parenchyma volume density (%)				
Tubular compartment	95.5 ± 0.7	96.6 ± 0.6	95.5 ± 0.4	96.3 ± 0.9
Tunica própria	3.1 ± 0.1	2.7 ± 0.1	3.5 ± 0.1	2.9 ± 0.1
Seminiferous epithelium	86.7 ± 1.1	86.1 ± 0.2	85.1 ± 0.6	87.1 ± 0.4
Lumen	5.7 ± 0.6	7.8 ± 0.2	6.9 ± 0.3	6.3 ± 0.3
Intertubular compartment	4.5 ± 0.3	3.4 ± 0.1	4.5 ± 0.3	3.7 ± 0.2
Leydig cell	2.0 ± 0.4	1.1 ± 0.1	1.6 ± 0.2	1.4 ± 0.1
Blood vessels	1.0 ± 0.3	1.0 ± 0.1	1.4 ± 0.3	1.0 ± 0.2
Lymphatic space	0.5 ± 0.2	0.3 ± 0.02	0.5 ± 0.1	0.4 ± 0.02
Others	1.0 ± 0.3	1.0 ± 0.04	1.0 ± 0.1	0.9 ± 0.08
Tunica albugínea (%)	4.1 ± 0.2	4.0 ± 0.6	3.8 ± 0.2	6.7 ± 0.2
Tubular diameter (μm)	245 ± 3	233 ± 3	246 ± 6	181 ± 5
Seminiferous epithelium height (μm)	92 ± 2	90 ± 2	86 ± 2	69 ± 1
Tubular length per gram of testis (meters)	20 ± 1	22 ± 1	21 ± 1	35 ± 3
Total tubular length per testis (meters)	5.7 ± 0.3	4.2 ± 0.6	4.7 ± 0.3	2.1 ± 0.1

Morphometric testicular parameters are also shown in Table 1. In all investigated species, the volume densities of seminiferous tubules and the seminiferous epithelium were very high and respectively in the range of ~95–97% and ~85–87%. In contrast, Leydig cells composed only 1–2% of the testicular parenchyma. In general, the values found for the tubular diameter (~235–245 um) and the seminiferous epithelium height (~85–90 um) were similar in *A*. *cursor*, *A*. *montensis*, and *N*. *Lasiurus*, whereas the values observed for these parameters were quite lower in *O*. *nigripes*, which presented almost 70% higher tubular length per gram of testis.

### Stages of the seminiferous epithelium cycle

**Fig 1:**
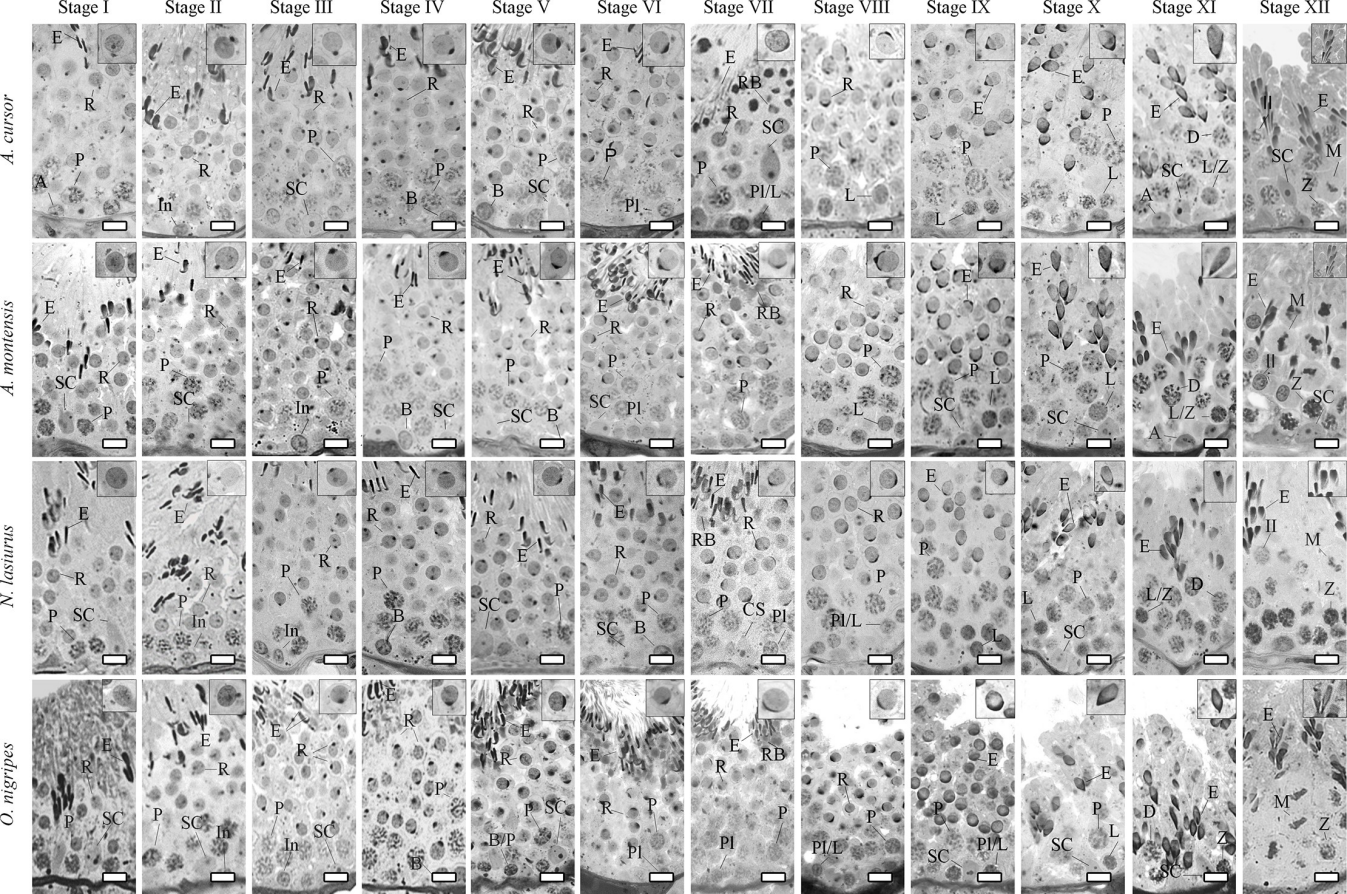
Different stages of the seminiferous epithelium cycle characterized according to the acrosome system in the four wild rodent species investigated. Twelve stages were characterized. The inserts in the upper right corner show a representative spermatid step present in each stage. Abbreviations: type A spermatogonia (A); intermediate spermatogonia (In); type B spermatogonia (B); pre-leptotene spermatocytes (Pl); pre-leptotene in the transition to leptotene spermatocytes (Pl/L); leptotene spermatocytes (L); leptotene in the transition to zygotene spermatocytes (L/Z); zygotene spermatocytes (Z); pachytene spermatocytes (P); diplotene spermatocytes (D); secondary spermatocytes (II); meiotic divisions (M); round spermatids (R); elongating/elongated spermatids (E); Sertoli cells (SC); and residual bodies (RB). Bar = 10μm.

For all rodent species herein investigated, twelve stages were characterized according to the acrosomic system and morphology of the developing spermatid nucleus (Fig 1). Also, spermiation occurred in stage VII and the germ cell morphology was quite similar in all species. The characterized stages are briefly described below.

#### Stage I

Two generations of spermatids were present in this stage: early round spermatids and elongated spermatids. Since the proacrosomal granules cannot be observed by light microscopy in this stage, the newly generated round spermatids were characterized by the absence of a visible acrosome vesicle.

#### Stage II

Early round spermatids usually present a tiny acrosomal vesicle containing acrosomal granules in contact with the nucleus. Elongated spermatids were located closer to the tubular lumen.

#### Stage III

Acrosomal vesicles flatten over the nucleus of round spermatids. These acrosomal vesicles formed angles of approximately 28 ± 2°, 29 ± 2°, 41 ± 3°, and 44 ± 2° over the nuclear surface respectively in *A*. *cursor*, *A*. *montensis*, *N*. *lasiurus* and *O*. *nigripes*. Elongated spermatids were observed in bundles.

#### Stage IV

In this stage, the angles of the acrosomal vesicles over the round spermatids nuclear surface were 33 ± 1°, 34 ± 1°, 44 ± 2°, and 54 ± 2° respectively in *A*. *cursor*, *A*. *montensis*, *N*. *lasiurus* and *O*. *nigripes*. Elongated spermatid bundles slightly moved towards the seminiferous tubule lumen.

#### Stage V

Elongated spermatids were located very close to the tubular lumen. The angles of acrosomal vesicles over the round spermatids nuclear surface were 41 ± 3°, 44 ± 3°, 54 ± 2°, and 57 ± 2° in *A*. *cursor*, *A*. *montensis*, *N*. *lasiurus* and *O*. *nigripes*, respectively.

#### Stage VI

Elongated spermatids were dissociated and located closer to the luminal border. The angles of acrosomal vesicles over the round spermatid nuclear surface were respectively 61 ± 4°, 61 ± 2°, 61 ± 3°, and 73 ± 2° in *A*. *cursor*, *A*. *montensis*, *N*. *lasiurus* and *O*. *nigripes*, respectively.

#### Stage VII

Elongated spermatids exhibiting sickle-shaped heads were located on the luminal border or undergoing spermiation towards the tubular lumen. Residual bodies were seen below these cells. Over the round spermatids nuclear surface, the angles formed by acrosomes were 97 ± 2°, 92 ± 4°, 78 ± 4°, and 83 ± 2° in *A*. *cursor*, *A*. *montensis*, *N*. *lasiurus* and *O*. *nigripes*, respectively.

#### Stage VIII

Since all elongated spermatids had spermiated, this stage had only one generation of spermatids. The angles of acrosomes over the round spermatids nuclear surface were 103 ± 3°, 102 ± 2°, 86 ± 4°, and 93 ± 4° in *A*. *cursor*, *A*. *montensis*, *N*. *lasiurus* and *O*. *nigripes*, respectively.

#### Stage IX

The nuclei of round spermatids started to elongate. The acrosomes also underwent an elongation process and formed angles over the nuclear surface of 105 ± 4°, 111 ± 2°, 95 ± 5°, and 106 ± 2° in *A*. *cursor*, *A*. *montensis*, *N*. *lasiurus* and *O*. *nigripes*, respectively.

#### Stage X

Nuclei of elongating spermatids started to form bundles. A ventral angle appeared in the elongating spermatid heads, and the ratio between the shortest and the longest longitudinal axis in *A*. *cursor*, *A*. *montensis*, *N*. *lasiurus* and *O*. *nigripes* was respectively 1.3 ± 0.1, 1.8 ± 0.4, 2.3 ± 0.1, and 1.5 ± 0.1. These nuclei also showed a polarization, and their heads were oriented toward the base of the tubule.

#### Stage XI

At this stage, spermatids completed their elongation process, and their nuclei were markedly grouped in bundles oriented toward Sertoli cell nuclei. The ratio between the shortest and the longest longitudinal axis in *A*. *cursor*, *A*. *montensis*, *N*. *lasiurus* and *O*. *nigripes* was 1.9 ± 0.2, 2.5 ± 0.1, 2.5 ± 0.1, and 1.8 ± 0.1, respectively.

#### Stage XII

The main feature of this stage was the presence of meiotic figures related to the first and the second meiotic divisions. Therefore, secondary spermatocytes were observed. Nuclei of elongated spermatids were more condensed. The ratio between the shortest and the longest longitudinal axis in *A*. *cursor*, *A*. *montensis*, *N*. *lasiurus* and *O*. *nigripes* was respectively 2.9 ± 0.2, 2.8 ± 0.2, 2.8 ± 0.1, and 2.5 ± 0.2.

### Stage frequencies

The mean percentages of each of the twelve stages characterized, as well as the frequencies of pre-meiotic (stages VIII-XI), meiotic (stage XII) and post-meiotic (stages I-VII) phases, are displayed in Table 2. Stages III and VII were the most prevalent (~15 to ~20%) in *A*. *cursor*, *A*. *montensis* and *N*. *lasiurus*, whereas the frequencies of many stages (II, IV, V, VIII, X, and XI) were in the range of ~4–9% in all four species studied. The frequencies of pre-meiotic and post-meiotic phases were similar among *A*. *cursor*, *A*. *montensis*, *N*. *lasiurus*, and *O*. *nigripes*, and in the range of respectively 28–33% and 61–64%.

**Table 2:** Relative frequencies (%) of the stages and phases of the seminiferous epithelium cycle in *A*. *cursor*, *A*. *montensis*, *N*. *Lasiurus* and *O*. *nigripes* (mean ± SEM).

Stages and phases of the seminiferous epithelium cycle			Species	
	*A*. *cursor*	*A*. *montensis*	*N*. *lasiurus*	*O*. *nigripes*
Stage I	4.2 ± 0.3	8.6 ± 1.4	4.6 ± 0.5	11.1 ± 1.2
Stage II	4.1 ± 0.4	6.4 ± 0.7	4.7 ± 0.3	6.1 ± 0.4
Stage III	15.7 ± 0.5	14.5 ± 1.1	16.3 ± 0.6	5.3 ± 0.7
Stage IV	6.3 ± 0.5	6.9 ± 1.5	6.1 ± 0.4	5.8 ± 0.1
Stage V	4.5 ± 0.5	4.0 ± 0.4	3.8 ± 0.4	7.5 ± 1.3
Stage VI	7.0 ± 1.0	6.5 ± 1.3	6.7 ± 0.6	12.9 ± 1.0
Stage VII	20.4 ± 0.9	17.5 ± 1.8	19.1 ± 0.8	10.9 ± 1.1
Stage VIII	7.1 ± 0.3	9.1 ± 1.0	6.5 ± 0.8	7.0 ± 1.1
Stage IX	12.6 ± 0.6	6.2 ± 0.9	12.7 ± 0.7	10.5 ± 0.8
Stage X	8.6 ± 0.9	5.9 ± 0.6	5.5 ± 0.6	6.3 ± 0.6
Stage XI	5.0 ± 0.6	6.2 ± 0.8	6.8 ± 0.7	6.5 ± 0.9
Stage XII	4.5 ± 0.4	8.2 ± 1.3	7.2 ± 0.7	10.1 ± 0.8
Pre-meiotic phase^1^	33.3 ± 1.9	27.4 ± 3.4	31.5 ± 2.8	30.3 ± 3.4
Meiotic phase^2^	4.5 ± 0.4	8.2 ± 1.3	7.2 ± 0.7	10.1 ± 0.8
Post-meiotic phase^3^	62.2 ± 3.4	64.4 ± 3.1	61.3 ± 3.7	59.6 ± 7.1

^1^After spermiation and prior to metaphase.

^2^ Meiosis I through meiosis II.

^3^After completion of meiosis until spermiation.

### Germ cell labeling and seminiferous epithelium cycle length

**Fig 2:**
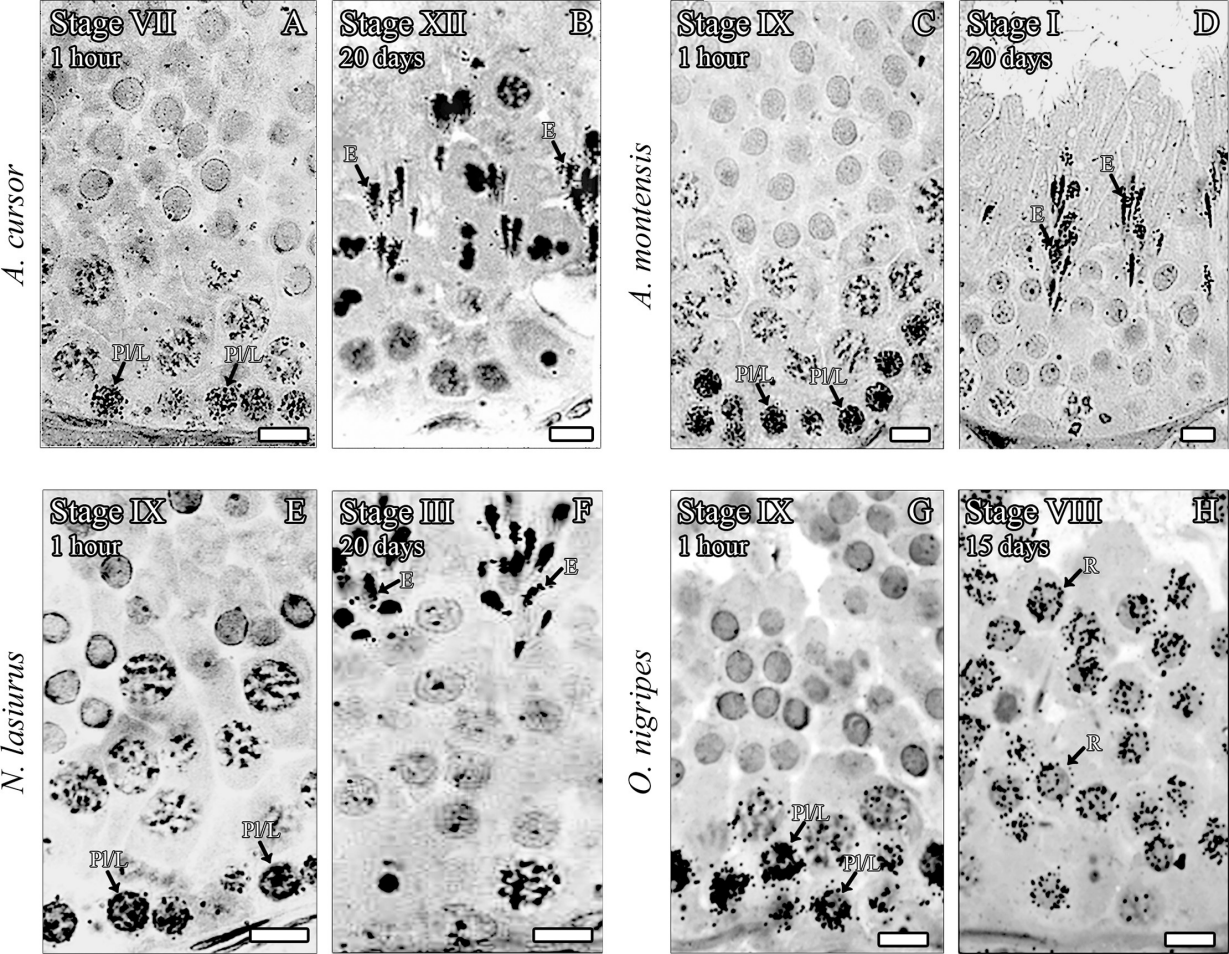
Evaluation of tritiated thymidine labeling. The most advanced germ cell type labeled (arrows) at different time periods (1 hour, 15 and 20 days) in *A*. *cursor* (A and B), *A*. *montensis* (C and D), *N*. *lasiurus* (E and F) and *O*. *nigripes* (G and H), after intratesticular tritiated thymidine injections. Bar = 10μm. Abbreviations: pre-leptotene in the transition to leptotene spermatocytes (Pl/L); round spermatids (R); and elongating/elongated spermatids (E).

As expected, approximately 1h after tritiated-thymidine injection, the most advanced labeled germ cells were identified as pre-leptotene in the transition to leptotene spermatocytes in all four species evaluated (Table 3 and Fig 2). These cells were found in the basal compartment of stage VII in *A*. *cursor* and stage IX in the other species (Table 3 and Fig 2).

**Table 3:** Length (days) of seminiferous epithelium cycle in *A*. *cursor*, *A*. *montensis*, *N*. *Lasiurus* and *O*. *nigripes* (mean ± SEM).

Species	Animal	Interval after	Most advanced germ	Stage of the	Number of cycles	Cycle
		Injection	cell type labeled	cycle	traversed	length
*A*. *cursor*	1	1 hour	Pl/L	VII	-	-
		19.8 days*	E	XII	2.33	8.49
	2	1 hour	Pl/L	VII	-	-
		19.8 days*	E	XII	2.37	8.35
	Mean duration of the cycle based on Pl/L = 8.42 ± 0.1 days.					
*A*. *montensis*	1	1 hour	Pl/L	IX	-	-
		21.8 days*	E	I	2.44	8.93
	2	1 hour	Pl/L	IX	-	-
		21.8 days*	E	I	2.46	8.86
	Mean duration of the cycle based on Pl/L = 8.89 ± 0.04 days.					
*N*. *lasiurus*	1	1 hour	Pl/L	IX	-	-
		20.1 days*	E	III	2.58	7.79
	2	1 hour	Pl/L	IX	-	-
		20.1 days*	E	III	2.57	7.82
	Mean duration of the cycle based on Pl/L = 7.8 ± 0.02 days.					
*O*. *nigripes*	1	1 hour	Pl/L	IX	-	-
		14.7 days*	R	VIII	1.83	8.00
	2	1 hour	Pl/L	IX	-	-
		14.6 days*	R	VIII	1.86	7.87
	Mean duration of the cycle based on Pl/L = 7.94 ± 0.1 days.					

*Time after thymidine injection minus 1 hour. Pl/L = preleptotene/leptotene spermatocytes; R = round spermatids; E = elongated spermatids.

Fifteen days after injection, round spermatids were the most advanced germ cell type labeled at stage VIII in *O*. *nigripes* (Table 3 and Fig 2), whereas elongated spermatids were the most advanced germ cell labeled in stage XII, stage I and stage III in *A*. *cursor*, *A*. *montensis* and *N*. *lasiurus*, respectively, after 20 days (Table 3 and Fig 2).

**Fig 3:**
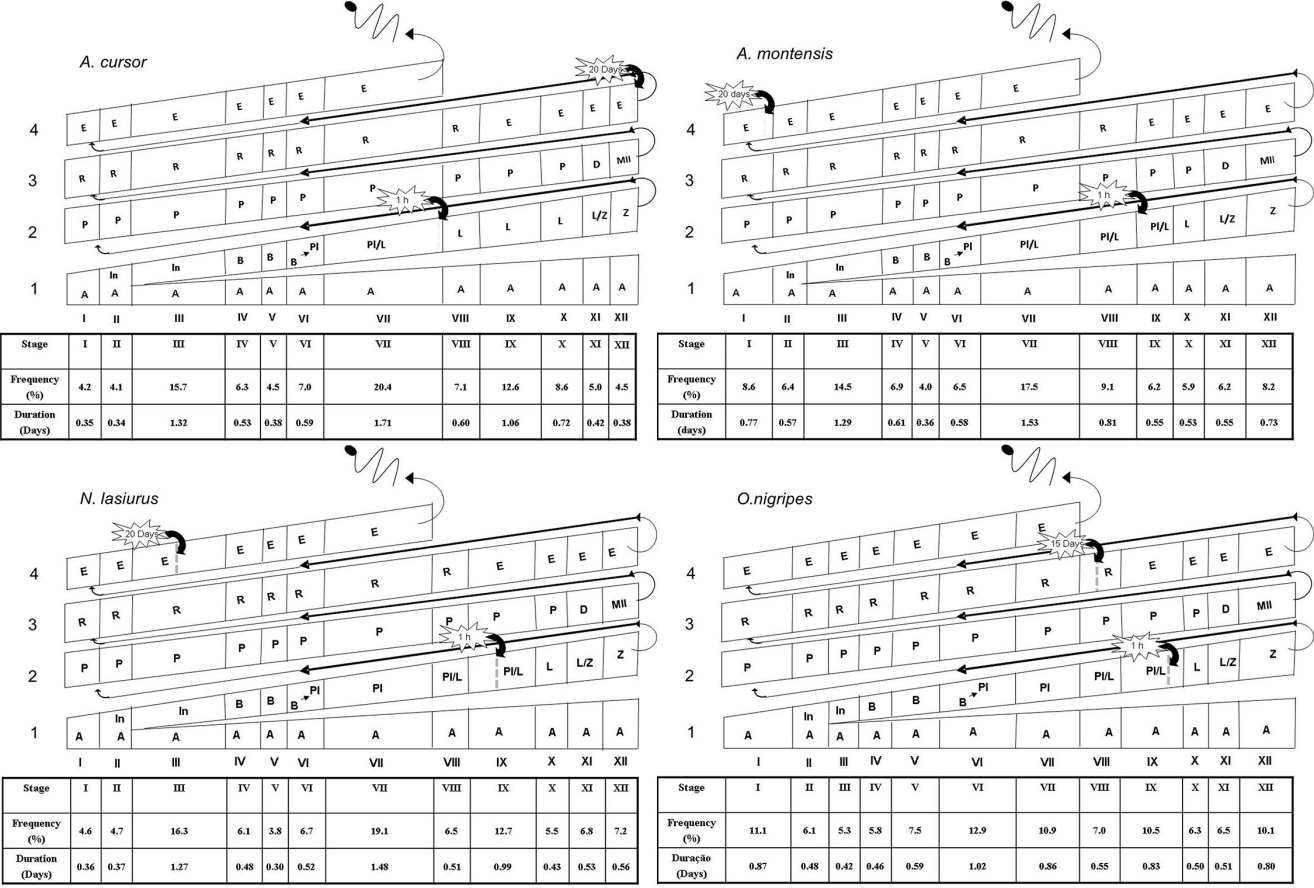
Schematic illustration of the frequencies (%) and duration (days) of the twelve stages of the seminiferous epithelium cycle characterized according to the development of the acrosome in the spermatids in *A*. *cursor*, *A*. *montensis*, *N*. *lasiurus* and *O*. *nigripes*. The most advanced germ cell labeled, one hour and 15 or 20 days after thymidine injection, is indicated by the thick arrow. The space corresponding to each stage of the cycle is proportional to its frequency and duration. Abbreviations: type A spermatogonia (A); intermediate spermatogonia (In); type B spermatogonia (B); pre-leptotene spermatocytes (Pl); pre-leptotene in the transition to leptotene spermatocytes (Pl/L); leptotene spermatocytes (L); leptotene in transition to zygotene spermatocytes (L/Z); zygotene spermatocytes (Z); pachytene spermatocytes (P); diplotene spermatocytes (D); secondary spermatocytes and meiotic divisions (MII); round spermatids (R); and elongating/elongated spermatids (E).

Based on the labeled germ cells observed in each period following thymidine injection and the stage frequencies (Tables 2 and 3 and Fig 2), the mean duration of the seminiferous epithelium cycle for *A*. *cursor*, *A*. *montensis*, *N*. *lasiurus* and *O*. *nigripes* was estimated, respectively as 8.4 ± 0.1, 8.9 ± 0.04, 7.8 ± 0.02 and 7.9 ± 0.1 days (Table 3). Since approximately 4.5 cycles are necessary for the entire spermatogenic process to be completed, the total length of spermatogenesis, also expressed in days, was estimated respectively as 37.9 ± 1.1 (*A*. *cursor*), 40 ± 0.9 (*A*. *montensis*), 35.1 ± 0.3 (*N*. *lasiurus*), and 35.6 ± 0.1 (*O*. *nigripes*). For each species, a schematic illustration of the stage frequencies and their duration is depicted in Fig 3.

### Cell counts and sperm production

The cell ratios and the Sertoli cell number and sperm production per gram of testis and testis are shown in Table 4. As observed in this table, except for *O*. *nigripes*, which presented a lower value, the meiotic index (number of round spermatids per primary spermatocyte) was approximately 3, meaning that the germ cell loss during meiosis was around 25%. Regarding the Sertoli cell efficiency (number of spermatids per Sertoli cell), *N*. *lasiurus* exhibited the highest value (~13:1), while *O*. *nigripes* showed the lowest efficiency (~8:1). The number of Sertoli cells per gram of testis in *O*. *nigripes* was almost 70 million, whereas in the other three rodent species the values found were in the range of ~48–55 million. Due to testis size, the total number of Sertoli cells per testis was ~4 million in *O*. *nigripes* and ~11–14 million in the other three species. In relation to the spermatogenic efficiency DSP/gram/testis, the values observed were ~62 million and ~66 million in *A*. *montensis* and *A*. *cursor*, respectively. This efficiency reached 74 million in *O*. *nigripes* and almost 80 million in *N*. *lasiurus*. Finally, the sperm production per testis was around 18 million in *A*. *cursor* and *N*. *lasiurus*, ~13 million in *A*. *montensis*, and less than 5 million in *O*. *nigripes* (Table 4).

**Table 4:** Cell ratios and sperm production in *A*. *cursor*, *A*. *montensis*, *N*. *lasiurus* and *O*. *nigripes* (mean ± SEM).

Parameter		Species		
	*A*. *cursor*	*A*. *montensis*	*N*. *lasiurus*	*O*. *nigripes*
Round spermatids: pachytene spermatocyte	2.9 ± 0.2	3.1 ± 0.3	3.0 ± 0.1	2.6 ± 0.1
Round spermatids: Sertoli cell nucleoli	10.9 ± 0.8	10.3 ± 0.7	13.2 ± 0.5	8.4 ± 0.2
Sertoli cell number per gram of testis (x 10 ^6^)	52.1 ± 4.3	54.7 ± 4.3	47.9 ± 3.7	69.2 ± 4.7
Sertoli cell number per testis (x 10 ^6^)	14.4 ± 1.6	11.3 ± 0.9	10.9 ± 8.1	4.1 ± 0.3
Daily sperm production per gram of testis (x 10 ^6^)	65.9 ± 3.8	62.0 ± 2.8	79.0 ± 5.1	74.0 ± 3
Daily sperm production per testis (x 10 ^6^)	18.5 ± 2.2	13.2 ± 0.9	18.1 ± 1.3	4.8 ± 0.4

### Leydig cell

All Leydig cell histomorphometric data are shown in Table 5. In comparison to the other three rodent species, *O*. *nigripes* showed the lowest individual Leydig cell volume (~400 um^3^ vs. ~800–1,100 um^3^). Because the Leydig cell individual volume is small in *O*. *nigripes*, overall, its number per testis gram (~33 million) was two-fold higher (~15–19 million) than in the three other species. However, as expected, its number per testis is much lower in *O*. *nigripes* (~2 vs. ~3–5 million; Table 5).

**Table 5:** Leydig cell morphometric parameters in *A*. *cursor*, *A*. *montensis*, *N*. *lasiurus* and *O*. *nigripes* (mean ± SEM).

Parameter		Species		
	*A*. *cursor*	*A*. *montensis*	*N*. *lasiurus*	*O*. *nigripes*
Nuclear diameter (μm)	8.0 ± 0.2	7.3 ± 0.1	7.6 ± 0.1	6.5 ± 0.1
Leydig cell volume (μm^3^)	1092 ± 131	807 ± 49	967 ± 21	393 ± 18
Nucleus volume (μm^3^)	275 ± 17	204 ± 11	235 ± 7	146 ± 4
Cytoplasm volume (μm^3^)	817 ± 115	603 ± 40	732 ± 52	247 ± 15
Leydig cell number per gram of testis (x10^6^)	19.4 ± 4.2	14.6 ± 1.8	17.1 ± 2.3	33.2 ± 2.9
Leydig cell number per testis (x10^6^)	5.2 ± 1.0	3.1 ± 0.5	4.0 ± 0.7	1.9 ± 0.1

## Discussion

Although rodents represent approximately 40% of all mammalian species alive, our knowledge regarding their reproductive biology is still incipient [19, 64–66]. In the present study, key testis morphofunctional parameters of four small rodent species from the Cricetidae family, inhabiting an ecotone of high biodiversity [14, 67], were evaluated. All four species herein studied exhibited high spermatogenic efficiency that resulted from a combination of several key testis functional parameters, such as the short duration of spermatogenesis and the very high values found for the percentage of seminiferous tubules in the testis parenchyma. The very high reproductive efficiency herein demonstrated can be a problem in an ecologically disturbed environment, leading to overpopulation of these four rodent species and potentially disseminating diseases among humans [2].

In general, several testis parameters were very similar among A. *cursor*, A. *montensis* and N. *lasiurus*. Interestingly, these species belong to the same clade, known as Akodontini [68]. However, O. nigripes, from the Oryzomyini clade [68], presented distinct features, such as reduced gonadossomatic index, lower Leydig cell size, higher incidence of apoptosis in the seminiferous epithelium and an increased number of Sertoli cell per testis gram. Nevertheless, to maintain high daily sperm production, the high Sertoli cell number observed for this particular species somehow compensated the germ cell loss.

In mammals, although under the control of the germ cell genotype [69], the length of the seminiferous epithelium cycle is not phylogenetically determined [53, 69–71] Nevertheless, similar to what has already been already described in fish [72], recent studies from our laboratory have shown that the duration of spermatogenesis in mice is accelerated by higher environmental temperatures [59]. For most mammalian species already investigated, the duration of one spermatogenic cycle is in the range of 9 to 12 days, and, overall, wild rodents have a faster spermatogenic cycle length [53, 60, 73, 74]. In fact, in the species herein evaluated, one spermatogenic cycle lasted only ~8–9 days and, taking into account that spermatogenesis in mammals requires approximately 4.5 cycles to be completed, *N*. *lasiurus* and *O*. *nigripes* exhibited one of the shortest durations of spermatogenesis (~35–36 days). An interesting aspect suggested in the literature, particularly in studies conducted in our laboratory investigating about twenty different species [38, 44, 75, 76], is the observation that the frequencies of pre-meiotic and post-meiotic phases of spermatogenesis are similar among phylogenetically closely related species, and in particular for those belonging to the same family. Therefore, confirming our hypothesis, in the present study, the four rodent species that belong to the Cricetidae family presented values following the pattern in which the post-meiotic frequency is markedly higher (see comparative Table 6).

**Table 6:** Comparative parameters related to biometry, testis stereology and spermatogenic events in well-investigated sexually mature rodent species from the Myomorpha and Hystricomorpha suborders.

				Suborder Myomorpha					Suborder Hystricomorpha	
	*A*. *cursor*	*A*. *montensis*	*N*. *lasiurus*	*O*. *nigripes*	Gerbil^a^	Hamster^b^	Rat^c^	Mouse^d^	*P*. *guyannensis*^e^	*T*. *moojeni*^*f*^
Body weight (g)	54	37	60	23	80	160	414	26–39	288	207
Testis weight (g)	0.29	0.22	0.24	0.06	0.54	1.7	1.57	0.095–0.113	1.63	0.97
Gonadosomatic Index (%)	1.07	1.1	0.8	0.5	0.72	2.13	0.76	0.76–0.55	1.15	0.93
Seminiferous tubules (%)	96	97	96	96	92	93	89	91–93	96	98
LC (%)	2	1.1	1.6	1.4	3	2.7	1.4	3.7–5.3	1.5	0,3
LC size (μm^3^)	1092	807	967	393	1148	1092	1207	1021–1450	746	799
LC number per gram of testis (10^6^)	19	15	17	33	28	55	13	29–49	19	4
Pre-meiotic phase^c^ (%)	33	28	32	30	36	26	24	22	49	40
Meiotic phase^d^ (%)	5	8	7	10	9	8	6	9	10	7
Post-meiotic phase^e^ (%)	62	64	61	61	55	67	71	69	41	53
Meiotic index^f^	2.9	3.1	3	2.6	2.8	3.3	3.4	2.3–2.8	2.7	3
Rate of apoptosis during meiosis	25%	22%	25%	35%	30%	17%	15%	43–30%	33%	26%
Spermatogenic cycle length (days)	8.42	8.89	7.8	7.94	10.6	8.7	12.9	8.6–8.9	7.5	8.6
Duration of spermatogenesis (days)	37.9	40	35.1	35.7	47.7	39.2	58	38.7–40	33.6	38.7
Sertoli cells per gram of testis (x10^6^)	52	55	48	69	28	45	27	39–41	78	53
Round spermatids per Sertoli cell	10.9	10.3	13.2	8.4	12.6	8.2	8.0–10.3	10.5–11.5	7.9	14.7
DSP per gram of testis (x10^6^)	66	62	79	74	33	24	17–24	45–48	78	82

Abbreviations: LC = Leydig cell; DSP = Daily Sperm Production.

References: [^a^^77^, ^b^^78^, ^b^^79^, ^c^^80^, ^c^^81^, ^c^^82^, ^d^^83^, ^d^^84^, ^e^^85^, ^f^^86^].

Similar to the mice strains usually utilized in laboratory studies (Table 6), the species from the Cricetidae family herein investigated presented low body weight and a gonadosomatic index considered relatively high for mammals [86], particularly in *A*. *cursor* and *A*. *montensis*. According to the literature [87], relative testis size and sperm morphology are indicators of the mating system, sexual behavior, and reproductive strategies related to sperm competition. Moreover, in order to survive, species with lower body weight are energetically more committed to reproduction [86]. In general, and in contrast to the Hystrichomorpha suborder, rodent species belonging to the Myomorpha suborder (including rats, hamsters and mice) are characterized by lower life expectancy, promiscuous mating system, multiple offspring per litter and per year and sperm with falciform heads instead of spatula shape [88, 89]. In fact, although there is still very little data available in the literature on the species herein investigated, the latter are known to be prolific and present more than one annual reproductive cycle [4]. Moreover, similar to the findings observed for other species from the Cricetidae family [90, 91], the qualitative and quantitative data herein obtained for all four species, such as spermatozoa with sickle-shaped/falciform heads and high DSP per testis gram, suggest that they might reproduce faster and efficiently in order to ensure their survival as a species.

In the present study, the percentage of the seminiferous tubules in the testis parenchyma was very high and similar to most rodents from the Myomorpha and Hystrichomorpha suborders investigated (see Table 6). It means that both Leydig cells’ volume density (%) and the number of these steroidogenic cells per testis gram are expected to be low, particularly compared to most mammalian species already investigated [44, 53, 60, 92].

We did not evaluate the individual Sertoli cell size and its occupancy (%) in the seminiferous epithelium in the present investigation. However, based on the Sertoli cell results herein obtained, such as their comparatively high number per testis gram and their good support capacity for germ cells (Table 6), according to the literature [73], we can assume that their individual size and consequently their occupancy in the seminiferous epithelium is not big. For instance, in mice, which in comparison to the four rodents herein investigated have close values for the seminiferous volume density, tubular diameter and Sertoli cell support capacity, the Sertoli cell percentage in the seminiferous tubule was the smallest (~14%) among the twelve mammalian species investigated [73]. Most importantly, in association with the short duration of spermatogenesis, the Sertoli cells findings herein obtained greatly accounted for the very high sperm production per testis gram (~60–80 million). To our knowledge, up-to-date this range of spermatogenic efficiency is only smaller than or similar to that observed in *P*. *guyannensis* and *T*. *moojeni* (~78–82 million) that belong to the Hystricomorpha suborder (Table 6). Also contributing to the very high spermatogenic efficiency, except for *O*. *nigripes*, the germ cell loss observed in our study was not expressive [53].

## Conclusion

In summary, in comparison to the other mammalian species already investigated and, in particular, with other rodents from the suborder Myomorpha, the four cricetid rodents herein evaluated exhibited very high spermatogenic efficiency. This efficiency is mainly related to the short duration of spermatogenesis, the high values found for the percentage of seminiferous tubules in the testis parenchyma and the number of Sertoli cells per testis gram. We expect that, besides contributing to comparative and evolutionary reproductive biology studies, the knowledge herein generated will help to advance the conservation programs and the proper management of wildlife.

## Supporting information

S1 Table Number of individuals per species collected in the rainy and dry seasons. (DOCX)
