# Supplementary material for: Testis structure, duration of spermatogenesis and daily sperm production in four wild cricetid rodent species (A. cursor, A. montensis, N. lasiurus, and O. nigripes)
Source: PLoS One. 2021 May 20;16(5):e0251256. doi: 10.1371/journal.pone.0251256 (PMC8136699; doi:10.1371/journal.pone.0251256)
Supplement: S1 Table — (DOCX) [file pone.0251256.s001.docx]

**S1 Table -** Number of individuals per species collected in the rainy and dry seasons.

| Species | Rainy season (Oct-Mar) | Dry season (Apr-Sep) |
| --- | --- | --- |
| 1. *Cursor* (n = 6) | 2 | 4 |
| 1. *Montensis* (n = 9) | 4 | 5 |
| *N. lasiurus* (n = 13) | 4 | 9 |
| *O. nigripes* (n = 11) | 4 | 7 |
